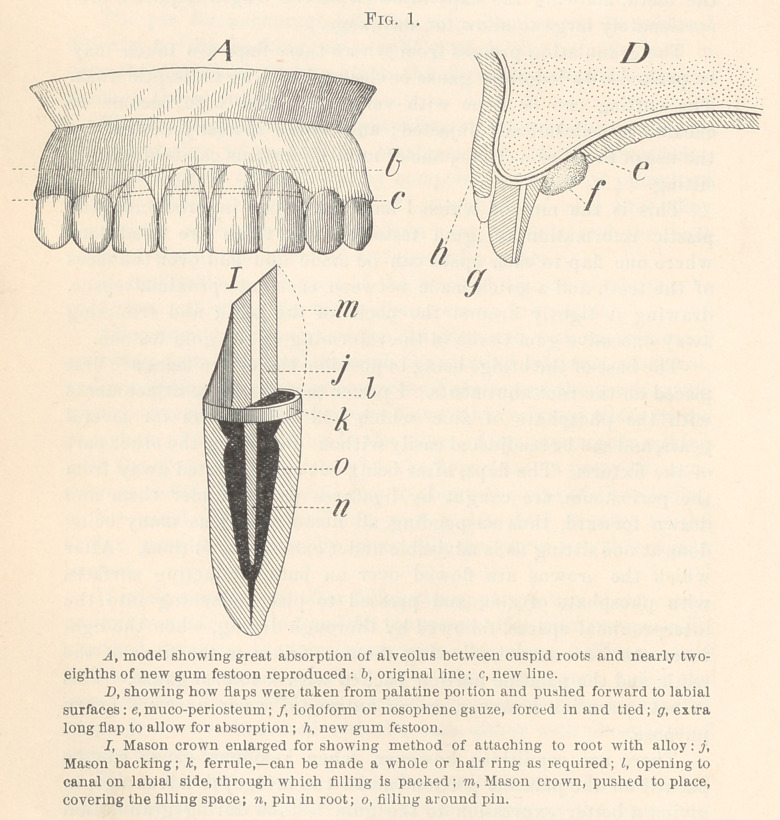# Some Features in Bridge-Work

**Published:** 1897-10

**Authors:** H. C. Register


### SOME FEATURES IN BRIDGE-WORK.[1]

BY H. C. REGISTER, M.D.

In Dr. M. L. Rhein's essay upon "Ideal Bridge-Work," read before the First District Dental Society of New York, December, 1893, he states, "At a time when mechanical skill in our laboratories was at its lowest ebb, one of the greatest operative dentists that ever lived, Marshall H. Webb, felt the need for something in the prosthetic line which would approach the ideal of excellence which he attained in his operative work. He took his cue from the work performed by Dr. B. J. Bing, of Paris, and first described in the *Dental Cosmos* of October, 1869, by Dr. H. D. Bennett, of Paris. Dr. Bennett's paper received little attention in this country until 1873, when Dr. Webb began the practice of inserting single artificial teeth into spaces, making anchorage in cavities in the approximal surfaces of the adjoining teeth. His method of work, as is well known, was a vast improvement on the style of that done in Paris.

Reports of isolated specimens of bridge-work are published as early as 1805 by J. B. Gariot, and in 1820 by C. I. Delabarre, and by Dr. S. S. Fitch, of New York, in 1829, and by Dr. W. H. Dwinelle in 1856. To Dr. B. J. Bing, however, is due the credit of reviving this antique method of inserting artificial teeth.

"A paper read by Dr. H. C. Register, entitled 'Grafting Artificial Crowns in Lieu of Plates,' before the Odontological Society of Pennsylvania, on January 8, 1881, shows that he had for some time been following Webb on this style of work."

This influx of new methods of inserting artificial teeth brought back to our dental laboratories the gold-workers, and private courses for mechanical dentists in plate-, bridge-, and crown-work were a source of considerable revenue to private instructors.

In this manner no one dares to contradict that bridge-work has been a great blessing to the profession. It has been the means of educating the men engaged in the mechanical construction of these devices to a point nearer to perfection in mechanical technique than has ever before existed in dentistry. It has also served as a stimulus to the inventive capacity of dentists, as is readily demonstrated by the vast number of methods of inserting artificial teeth without plates, which are now practised.

---

[1] Read at a meeting of the Academy of Stomatology, April 27, 1897.

In this relation I wish to say a few words in further advancing this really great possibility in prosthetic work. Dr. Webb, in discussing my paper in 1881, believed it possible to attach more than one tooth successfully, although he had never performed that operation himself.

In modern bridge-work there are two essentials,—namely, perfect retaining device upon the root attachments, and perfect cleansing surfaces. These two conditions are essential to the future usefulness of the fixture. Dr. Rhein criticises the so-called self-cleansing spaces generally made, as not only receptacles for *débris*, but as causing constant annoyance to the tongue and interference with speech.

I say with pleasure that I have never placed a bridge with these *cul-de-sacs*, always adhering to the principle of restoring lost anatomical forms.

To these two previous essential conditions I would add a third, applicable to the immediate oral teeth in their artistic relations. I desire to call your attention to the uncertainty of the present method of attaching bridges and crowns, especially those forms where several anchorages are utilized.

Phosphate of zinc is the agent in general use, and where the fixture contains several abutments its application must be made so rapidly that without intelligent assistance there is difficulty in properly adjusting it. It is a matter of considerable concern, after a beautiful piece of mechanical work is ready to be placed in position, whether we succeed or fail in making everything perfectly secure, and carry the bridge to a proper adjustment. To do away with this apprehension of failure, time should be at the disposal of the operator, and for greater artistic results a *better* crown can be made use of. To get time one attachment should be fixed, and the others following it in rotation, under conditions that the cementing or connecting process should be of such a character and disposition to resist to the greatest extent bacterial invasion.

We might say of phosphate of zinc, with our present knowledge of incipient caries, that it is a preventive of bacterial intrusion, less its comparatively rapid dissolution. This contra-disposition, in connection with its lack of strength when stress is applied, makes it non-dependable for carrying a bridge many years, unless the fixtures be so accurately made as to be self-supporting. In combination, however, with an alloy freshly cut and of good test, thoroughly mixed together, we have almost an ideal preserver of tooth tissue, less its inartistic appearance,—a material into which is worked a matrix that is mutually self-sustaining, both for filling and anchoring bridges or crowns. To apply this retainer in bridge- or crown-work the porcelain face or veneer should be of special form and appropriately made, so as to allow not only of manipulation but of artistic construction as well.

The filling of root-canals with alloy, and the foregoing combination of it and phosphate of zinc, is the method that has proved most successful in my hands. To apply a retainer of this character, the filling must be worked in by manipulation, either on bridge or crown, and the supporting base must be appropriately made to permit the filling-material being placed with confidence, and the artistic relations fully carried out in the work.

I made use of a crown that contained an inverted dovetailed groove placed on the back of the porcelain faces of the cuspids and incisors and the interproximal spaces of the bicuspids and molars.

One of the principal features in crowns of this character is in allowing the root to be filled from the labial or buccal in contradistinction to the palatine side, and of placing the supporting fixture before the porcelain faces are arranged in place, filling each root separately under perfect observation and without hurry, and in case of accidents these crowns are easily duplicated without damage to the fixture.

These teeth between the abutments were placed upon a saddle exactly their own diameter, which rested upon the alveolar ridge, the necks being enclosed in a ferrule for the bicuspids and molars, and in the immediate oral teeth in a half or whole ferrule as best suited the artistic result to meet the individual case. You will thus notice in the illustration, if at all familiar with the Mason crown, recently placed upon the market, and which opens a great possibility in prosthetic bridge- and crown-work, that they include the same means of attachment so far as the principle is involved.[1]

This method in no way bears upon the ingenious method adopted by Drs. Rhein and Andrews of placing split shells over inverted crowns and fixing them to place with a key.

The principle involved here is in the use of the roots of the teeth, hermetically sealing the same under observation, gaining greater strength in stress, and protecting the underlying tooth from carious influences.

---

[1] The principle involved, and the type of crown employed in the Mason adjustable crown, have been used by Dr. Rhein for several years. He states that the device was suggested to him by Dr. Van Woert.—(H. W. B.)

You will notice, if you are at all familiar with the Mason crown, that the face slips off and on the backing to the diameter of about half of the face of the root. Thus in placing the base of either crown or bridge into position, and the porcelain face removed, you have half the root exposed by simply opening up a floored ferrule, and thus exposing the pulp-chambers. Where half ferrules are used the same result, of course, is gained.

The pin for anchorage into the pulp-chamber can be soldered directly to the backing, or a split post, with the ends bent in opposite directions resting upon the ferrule, packed to position through this opening.

Where amalgam is used slight oiling of the gold should precede the adaptation to prevent its amalgamation. Everything being ready, the parts dried, a creamy compound is made of phosphate of zinc alone, or equal parts of phosphate of zinc and alloy thoroughly rubbed together under a heavy spatula, and the inner edges of the ferrule anointed and pressed to place.

If preferable, in this part of the work phosphate of zinc can be used alone; this is to fix the bridge or crown to position.

After drying that part of the retainer and with an excavator removing from the canal through the opening into root all excess, and desiccating by means of compressed air, repeat the combination mixture, except to make the second and other mixings stiffer in the phosphate of zinc and drier in the alloy. After thoroughly mixing with the spatula manipulate with the fingers into a putty-like consistence, rolling it in the fingers to about the size of the opening in the canal. If the retaining post is attached directly to the bridge, pass it in piece by piece till the parts are thoroughly packed.

If using a split post, after filling the upper portion of the canal, press it home to position, and fill the same as if it were permanently attached, finishing the last layers with alloy alone. Where a half ferrule is made, this is carried to the edge of the root under the gum festoon. The tooth crown, which has previously been properly adapted, is now connected with the base either with phosphate of zinc or chloro-percha.

Where it is desirable to resist the greatest stress I recommend the use of alloy alone, packed in the same way as the preceding mixture.

In spaces between the abutments, where resorption has followed the extraction of teeth, there is characteristic tooth and gum contact which gives an artificial appearance to the facial expression.

To overcome this artificial appearance it is necessary to reproduce the gum festoon. The reforming of this on the faces of the teeth consists in taking the mucous and submucous tissue, with some fibre, from the palatial or lingual portion of the jaws in the form of flaps cut in festoons as wide as the teeth where they join at the interproximal spaces, and stretching it over the faces of the teeth, allowing the flaps to be twice the length required, proportionately large to allow for shrinkage.

The granulating process from where these flaps are taken may be packed with iodoform gauze or cleansed with an antiseptic wash. The cutting can be done with very little pain with cocaine or eucaine hypodermically injected; and where specially desired by the use of the Mason crown, one or more operations can be done at a sitting.

This is the method which I have employed in producing the plastic reformation of gum festoon. But there are conditions where one flap to each space can be made and laid over the faces of the teeth and a stitch made between each interproximal space, drawing it tightly around the necks of the teeth and trimming away excessive gum tissue in the reforming of the gum festoon.

The base of the bridge being in position, the crown faces are first placed on the root abutments. I prefer to make these attachments with the phosphate of zinc, which will remain firm for several years, and can be readjusted easily without disturbing the other part of the fixture. The flaps, after being cut and dissected away from the periosteum, are caught by ligatures passed under them and drawn forward, thus suspending all hemorrhage; as many being done at one sitting as is advisable under existing conditions. After which the crowns are flowed over on both contacting surfaces with phosphate of zinc and pressed to place, pinching into the interproximal spaces, followed by thorough drying, when the ligatures can be removed, allowing them to fall over the faces of the teeth, and the palatine portion packed with iodoform gauze. This is not essential, however, as an antiseptic wash answers every purpose.

There are conditions, however, when iodoform gauze can be packed on the palatine portion (under the bridge) to advantage, in giving a better expression to the gum festoon during granulation by causing it to bulge.

In a case performed by myself last winter, a most gratifying result was secured for the four incisors attached to the cuspid roots, where a crescent-shaped arch was caused by recession, the centrals being fully twice the length of the cuspids. This discrepancy, which was very unsightly on account of the incisors having to be so much longer, was wholly overcome by using long flaps over the centrals, and shorter ones over the laterals, reproducing the gum on a line and festoon between the abutments. This is illustrated in Fig. 1.

I saw this patient several months after operating, and the gum presented nearly a perfect line between the cuspids, and the granulation which had followed the filling in of the spaces from where the flaps were taken presented a perfectly healthy and normal appearance. A singular and gratifying result from this operation, in connection with the plastic work, was a perfect tightening of the roots, which before presented considerable luxation.

With that class of patients showing the gum-line of tooth contact this plastic operation of gum-formation opens up a new line of possibilities in giving most pleasing results, which I feel sure will find some commendation in artistic bridge-work.

## Figures and Tables

**Fig. 1. f1:**